# Effects of quinpirole in the ventral tegmental area on impulsive behaviour during performance on the five-choice serial reaction time task

**DOI:** 10.1007/s00221-022-06502-8

**Published:** 2023-01-10

**Authors:** Chiara Toschi, Trevor W. Robbins, Jeffrey W. Dalley

**Affiliations:** 1 Department of Psychology and Behavioural and Clinical Neuroscience Institute, University of Cambridge, Downing St, Cambridge, CB2 3EB UK; 2 Department of Psychiatry, Hershel Smith Building for Brain and Mind Sciences, Addenbrooke’s Hospital, University of Cambridge, Cambridge, CB2 0SZ UK

**Keywords:** 5CSRTT, Waiting impulsivity, Quinpirole, D2/3 receptors, Premature responding, Dopamine

## Abstract

Impulsive behaviour on the five-choice serial reaction time task (5CSRTT), a task measuring attention and impulsivity in rodents, is known to depend on dopamine (DA) neurotransmission in the mesolimbic DA pathway. Previous research in our lab reported that systemic administration of the D2/3 agonist quinpirole, which decreases DA release in the striatum, reduced premature responses in rats performing the 5CSRTT. It is unclear, however, whether this effect is mediated by the activation of inhibitory somatodendritic receptors in the ventral tegmental area (VTA), which in turn leads to a reduction in DA release in the nucleus accumbens, a major terminal region of the mesolimbic DA pathway. In the present study, we investigated this possibility by infusing quinpirole directly into the VTA of rats during performance on the 5CSRTT. We found that quinpirole, at the highest dose, significantly reduced the frequency of premature responses on the 5CSRTT. Thus, the effects of quinpirole and other D2/3 receptor agonists to reduce this form of impulsive behaviour appear to depend on the activation of somatodendritic D2/3 receptors in the VTA.

**Supplementary Information:**

The online version contains supplementary material available at 10.1007/s00221-022-06502-8.

## Introduction

Impulsivity is a multidimensional trait in humans and other mammalian species and underlies psychiatric disorders such as Attention-Deficit/Hyperactivity Disorder (ADHD, Vassileva and Conrod 2019), substance use disorder (SUD, Dalley and Robbins 2017; Jentsch and Taylor 1999) and depression (Swann et al. 2008). Different taxonomies have been advanced regarding the different sub-types of impulsivity (Bari and Robbins 2013). Generally speaking, however, impulsivity has often been divided between ‘motor impulsivity’ (or impulsive action) and ‘choice impulsivity’ (or impulsive choice). While the former refers to deficits in motor inhibition and the inability to withhold a prepotent dominant response, the latter refers to deficits in reward-based responding and the propensity to choose a small, immediate reward over a larger but delayed reward (Bari and Robbins 2013; Eben et al. 2020; van Gaalen et al. 2006). Waiting impulsivity, has been shown to confer vulnerabilities to substance abuse both in humans and in animals (Belin et al. 2008; Dalley et al. 2007; Diergaarde et al. 2008; Marusich and Bardo 2009; Oberlin and Grahame 2009; Poulos et al. 1995; Radwanska and Kaczmarek 2012; Sanchez-Roige et al. 2014) and for this reason has received much attention in recent years.

Waiting impulsivity is defined as the inability to withhold a motoric response for a specified period, even if this leads to negative consequences. One task that has been widely used to study this behaviour in animals is the five-choice serial reaction time task (5-CSRTT, Robbins 2002). Here, animals are trained to detect a visual cue (i.e., a brief appearance of light) in one of five spatially distinct apertures in an operant chamber. Responses before the onset of the visual target cue (i.e., a premature response) are signalled with a 5 s time-out and reward omission. This task has been adapted to human participants and it has been shown to be sensitive to increased impulsiveness in SUD patients (Voon et al. 2014), similarly to what is observed in rodents with an innate vulnerability to develop cocaine addiction-like behaviours (Dalley et al. 2007; Belin et al. 2008).

Substantial research has investigated the neural processes contributing to premature responding (for a review see Dalley and Robbins 2017). Early evidence pointed to a role of the mesolimbic dopamine (DA) pathway in the manifestation of this behaviour. For example, it was shown that systemic administration of the indirect DA agonist, d-amphetamine, increased premature responding; an effect that was blocked by selective depletion of DA within the nucleus accumbens (NAcb, Cole and Robbins 1989) and intra-accumbens infusions of a D2 receptor antagonist (Pattij et al. 2007). In line with this evidence, trait impulsivity in rodents, as assessed on the 5-CSRTT, was associated with reduced density of the DA transporter (DAT) and DA D2/3 receptors in the shell sub-region of the NAcb (Dalley et al. 2007; Jupp et al. 2013). Under the assumption that abnormalities in the level of DA D2/3 receptors affect primarily autoreceptors located on DA fibres projecting to the NAcb, these findings have led to the hypothesis that impulsive responding on the 5CSRTT arises from increased dopaminergic release from VTA terminals into the NAcb shell (Dalley and Robbins 2017). This hypothesis is supported by findings in rodents that HI rats have reduced mRNA levels of DA D2 receptors in the VTA (Besson et al. 2013).

To further investigate whether trait-like impulsivity is mediated by a hyper-dopaminergic state in the VTA-NAcb circuit, we tested whether pharmacologically reducing the activity of DA cells in the VTA, and consequently in the striatum, reduces premature responding. Prior research in the laboratory established that systemic administration of the selective D2/3 agonist quinpirole diminishes impulsivity in a dose-dependent manner (Fernando et al. 2012), however the neural locus of this effect has not been established; DA D2/3 receptors are richly expressed in several terminal regions of the mesolimbic DA system, including the dorsal and ventral striatum, as well as on the soma and dendrites of DA cells in the VTA (Dalley and Everitt 2009). This ubiquitous receptor is expressed presynaptically on DA neurons to regulate DA neuronal excitability (via somatodendritic autoceptors) and DA release in terminal fields (via inhibitory autoreceptors). This receptor is also expressed postsynaptically to mediate DA neurotransmission, most notably the indirect pathway of the basal ganglia (Cox et al. 2015). D2/D3 receptors also act as heteroreceptors involved in regulating the release of neurotransmitters such as glutamate and acetylcholine in the striatum (Marchi and Grilli 2010). In the present study, quinpirole was infused directly in the VTA to restrict the pharmacological activity of this D2/D3 agonist to inhibitory somatodendritic receptors. Based on electrophysiological evidence (White and Wang 1984), we predicted that quinpirole would suppress the activity of DA neurons in the VTA and cause a reduction in DA release within terminal regions of the mesolimbic DA system, including the NAcb. We expected that this would, in turn, reduce premature responses.

## Materials and methods

### Animals

Twelve outbred male Lister Hooded rats (Charles River, Margate, UK) weighing 280–300 g at the beginning of the experiments were used. Animals were acclimatised to the animal facility under a 12 h:12 h light cycle (lights off at 7 AM) for a minimum of 7 days before any procedure began. When rats reached a body weight of approximately 300 g, they were food-restricted to maintain approximately 90% of their free-feeding weight trajectory (19 g of Purina rodent chow per animal and day; adjusted for reward pellet consumption during testing). Water was available ad libitum and food was given at the end of each day’s testing. All procedures conformed to the UK (1986) Animal (Scientific Procedures) Act (Project licence 70/7548 and PA9FBFA9F: Neurobehavioural mechanisms of mental health, held by Dr. A. L. Milton) and were approved by the local Ethics Committee at Cambridge University.

### Five-choice serial reaction time task: training and screening for impulsivity

#### 5-CSRTT apparatus

Twelve five-hole operant chambers (Med Associates, Georgia, VT) controlled by two computers and Whisker Control software (Cardinal and Aitken 2010) were used. Each chamber was enclosed in a ventilated sound-attenuating box, fitted with five apertures in a curved wall and a food magazine on the opposite wall of the box that delivered rodent sugar pellets (TestDiet®, Purina, UK). A yellow light-emitting diode stimulus was placed at the rear of each aperture. The food magazine and the entire chamber was illuminated by light emitting diodes. Infrared beams detected responses in the magazine and apertures.

#### Training

All rats were trained on the 5CSRTT as described previously (Bari et al. 2008). Animals were trained to detect a brief visual cue appearing in one of five apertures of the operant chambers. Each trial is initiated when the rat pokes into the food magazine and the visual cue is presented after an ITI of 5 s. A response was deemed ‘correct’ if the animal poked into the hole where the light was presented within 5 s of target presentation. A nose-poke response occurring before the appearance of the visual cue was considered ‘premature’, while a response occurring in any of the apertures where the light was not presented was considered ‘incorrect’. A failure to respond within 5 s of target presentation was recorded as an ‘omission’ of response. Only correct responses were rewarded with a food pellet (Noyes dustless pellets, Research Diets, UK), while incorrect, premature and omission responses were punished with a time-out period of 5 s. During a time-out, the animal was required to wait for the beginning of the next trial to engage again with the task. Nose-pokes in any of the holes made after a correct or incorrect response, but prior to reward collection, were deemed ‘perseverative’ but were not signalled by punishment. Each session lasted a maximum of 100 trials or 30 min, whichever limit was reached first. During the training session, stimulus duration was set at 30 s and was gradually decreased over sessions until animals reached stable baseline performance (accuracy, > 80% correct choice and < 20% errors of omission). Rats were kept in the training phase until they reached a stable baseline performance with a final stimulus duration of 0.7 s and an ITI of 5 s. Rats were subsequently exposed to three fixed 7 s ITI sessions, each separated by two days of baseline testing. This was done with the aim to expose animals to the long ITI challenges before the intra-VTA pharmacological manipulations. We decided to test our quinpirole manipulations during sessions with a long 7 s ITI challenge because this induces more premature responses thus allowing for more subtle effects of the drug to emerge.

### Intracranial surgery

Surgical procedures were performed following standard stereotaxic techniques. For all surgeries, rats were anaesthetised using isofluorane in 5% oxygen and secured in a stereotaxic frame fitted with atraumatic ear bars. Anaesthesia was generally maintained at 2.5–3% isoflurane. Baytril (1 mg/kg; 100 mg/ml; Bayer, Germany) and Metacam (1 mg/kg; 5 mg/ml; Boehringer Ingelheim, Germany) diluted in distilled water 1:1 were injected subcutaneously prior to surgery. Bilateral 22-gauge double guide cannulae (Plastics One, Sevenoaks, UK), extending 4 mm below the plastic pedestal, were implanted bilaterally above the VTA (coordinates in mm relative to Bregma: AP. -5.4; ML. 0.75. DV. -1.6 (below dura); Cannulae were secured to the skull with dental acrylic and stainless steel screws and occluded by a stylet and a dust cap. After surgery, animals recovered for 7 days in their home cages (single-housed).

### Drugs

(−)-Quinpirole hydrochloride was purchased from Sigma (St. Louis, MO, USA), dissolved in filtered 0.9% saline, and administered by intracranial infusion (0.25 μl per infusion at a rate of 0.125 μl/min). The range of doses was chosen to mimic that used by Fernando and colleagues (2012). The concentration of doses was adapted to be suitable for intracranial infusion and had been previously tested in the lab for other experiments (Moreno et al. 2013).

### Intracranial microinfusions

Drug and vehicle infusions were given 12 min before behavioural testing. Micro-infusions were delivered through a 28-gauge bilateral injector (Plastics One, Roanoke, USA), inserted through the guide cannula and extending 6.5 mm beyond the tip of the guide. Animals were habituated to the infusion procedure over two daily sessions separated by a day of just baseline training on the 5CSRTT (5 s ITI). On the first habituation day, the injector was lowered into the double guide cannula and left in place for 1 min. On the second habituation day, rats received a single vehicle infusion over 2 min (saline, 0.25 μl) and were then run on the 5CSRTT as a normal baseline training day (5 s ITI). During the infusion procedure, rats were gently restrained by the experimenter while the obturators were removed from the cannulae and the injectors lowered into the intended brain region. Prior to, and after each infusion, the injector remained in the brain for 1 min. When the injector was removed, the obturator was cleaned with ethanol (2%), rinsed in distilled deionised water, and lowered through the guide cannula. The animal was then placed into the test apparatus.

Following re-establishment of stable performance on the 5CSRTT, intracerebral microinfusions of quinpirole were carried out. Infusions of quinpirole (veh, 0.01, 0.03, 0.3 and 1 ug/ul) were delivered according to a randomized Latin-square design and were tested with sessions comprising a long ITI of 7 s. The infusion experiments were run over a 3-day cycle, starting with an initial baseline session (5 s ITI). On day 2, animals received an infusion of drug or vehicle (veh) before testing on a long ITI session (ITI: 7 s; SD: 0.7 s). On day 3, animals were tested again on a baseline session (5 s ITI; SD: 0.7 s).

### Histological verification of cannulae placements

Rats were anaesthetised with an overdose of sodium pentobarbital and transcardially perfused with saline followed by 10% buffered formalin. The brain was removed and stored for at least 48 h in a 30% sucrose solution. The brain was sectioned using a Leica CR cryostat (chamber temperature: − 19 °C; sample temperature: − 18 °C) and coronal Sects. (60 μm) were collected across the whole brain. Every sixth section was mounted on glass slides and stained with Cresyl Violet. The sections were used to verify cannulae tip placement.

### Data analysis

The main variables of interest across experiments were the number of reinforcers earned; accuracy; number of premature responses; number of omissions; response latency.

Statistical tests were performed with RStudio, version 1.2.1335 (RStudio, Inc). Data were subjected to Linear Mixed-Effects Model (LMEM) analysis with the lmer package in R. In all experiments, percentages or probabilities were arcsine square root transformed, integer numbers (e.g., number of reinforcers earned) were square root transformed. Latencies were log-transformed. Transformations were applied to avoid incurring issues of non-normal data distributions. Accuracy was always calculated as following: number of correct responses/(correct + incorrect responses). To validate whether the data transformations improved model fit we compared the Akaike information criterion (AIC) values of the models with transformed and non-transformed data. The model with transformed data yielded the lowest AIC values for all variables. All models with a within-subject factor had the factor ‘subject’ modelled as a random slope to account for individual differences between rats across testing sessions. When significant three-way interactions were found, further analysis was performed by conducting separate multilevel models on a specific variable of interest. For all analyses, significance was considered at *α* = 0.05. When significant interactions were found, further analysis was performed by conducting post hoc Tukey’s corrected pairwise comparisons. For drug manipulations post hoc testing was used to determine differences with vehicle treatment only. Coefficients representing the change in number of premature responses following treatment, for each animal, were obtained by fitting a simple linear regression. A Pearson linear correlation was then used to look for a relationship between these coefficient values and premature responses made during treatment with vehicle. The datasets generated and analysed during the current study are available from the corresponding author on reasonable request.

## Results

### Histology

**Fig. 1:**
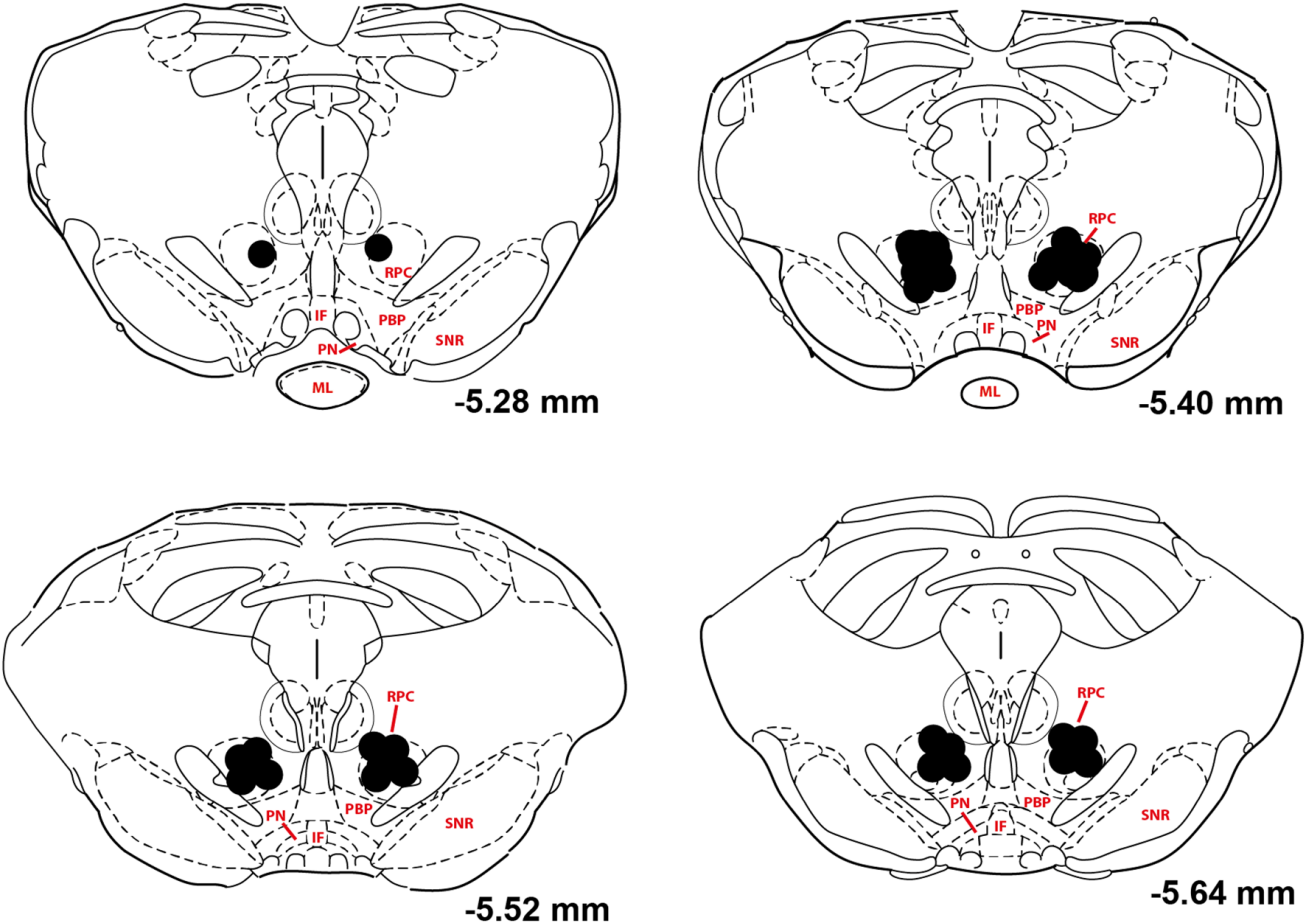
Injector tip placements in the VTA. *PN*  paranigral nucleus of the VTA; *IF*  interfascicular nucleus; *ML*  medial mammillary nucleus, lateral part; *PBP*  parabrachial pigmented nucleus of the VTA; *SNR*  substantia nigra, reticular part; *RPC*  red nucleus, parvicellular part (Paxinos and Watson 2007)

The ventral-most locations of injectors are included in Fig. 1. No rats were excluded from the study. For an exemplar image of a VTA cannulation see the supplementary materials (Fig. S1).

### Effects of microinfusions of quinpirole into VTA during performance on 5CSRTT

**Fig. 2:**
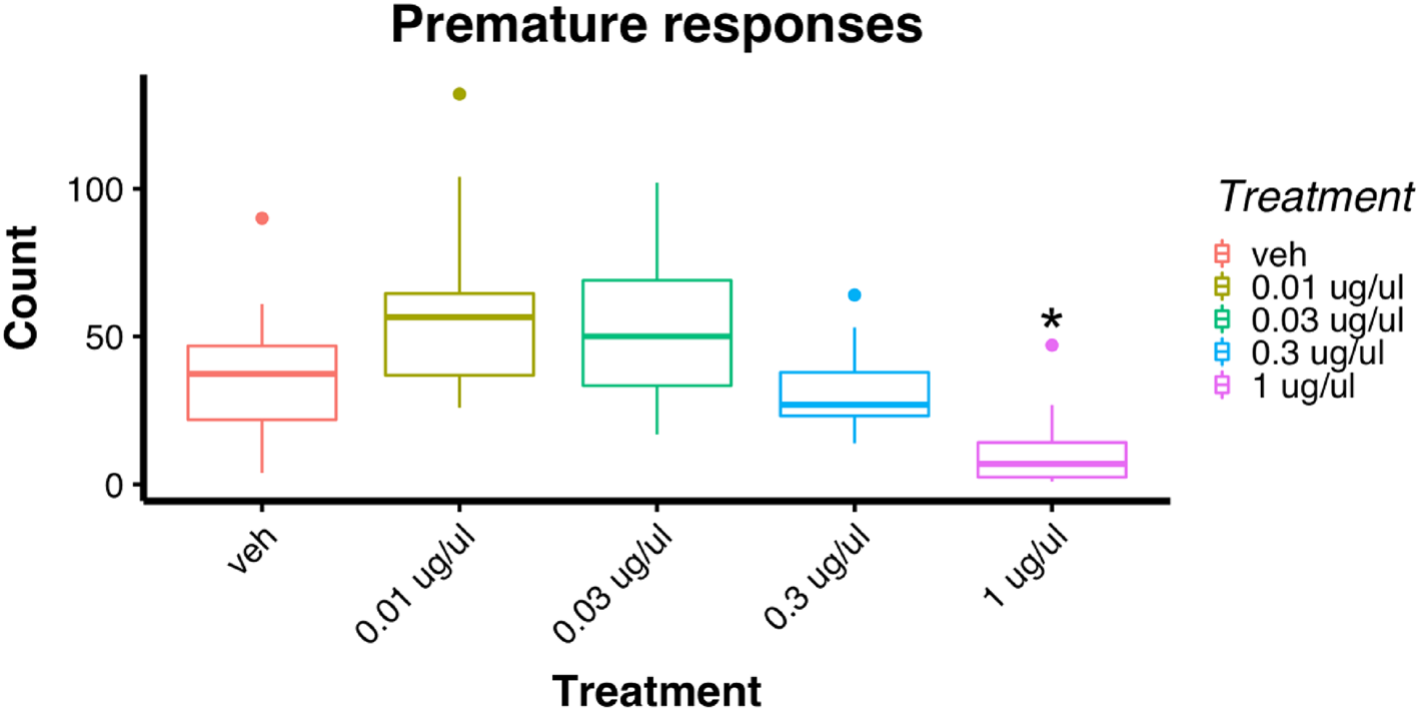
Effects of intra-VTA infusions of quinpirole on premature responses in the 5CSRTT. *Significant difference compared with vehicle *p* < 0.05

There were no significant main effects of quinpirole dose on the number reinforcers earned [*F*(4,44) = 1.12, *p* = 0.359] or accuracy [*F*(4,44) = 1.42, *p* = 0.243]. As shown in Fig. 2, however, quinpirole did affect the number of premature responses [*F*(4,44) = 14.98, *p* < 0.001], with the highest dose significantly decreasing premature responses compared with the vehicle condition and intermediate doses (*p* < 0.05 for all comparisons). The highest dose also produced more omissions than the vehicle and all other doses, however this difference did not reach significance (*F*(4,44) = 1.90, *p* = 0.127, see Table 1). Table 1 also summarises results for collection and response latencies. Briefly, after administration of the highest dose of quinpirole rats were slower at collecting food and responding to the cue in the front panel of the 5-CSRTT chamber. There was a significant negative correlation between the number of premature responses at vehicle and the change in number of premature responses following quinpirole treatment [*r* = − 0.84, *p* < 0.001, see Fig. S2].

**Table 1:** Omission responses and response latencies on the 5CSRTT following intra-VTA infusions of quinpirole (0, 0.01, 0.03, 0.3, 1 μg/μl)

Variable	Vehicle	0.01 μg/μl	0.03 μg/μl	0.3 μg/μl	1 μg/μl
Omissions	9.05 (2.03)	8.68 (1.99)	9.98 (2.14)	8.09 (1.92)	14.17 (2.54)
Correct latency (ms)	675.17 (35.15)	646.10 (31.98)	668.22 (41.20)	682.46 (43.39)	818.05* (55.97)
Incorrect latency (ms)	1405.78 (156.59)	1205.24 (120.86)	1226.74 (132.77)	1380.94 (99.84)	1863.14* (188.04)
Collection latency (ms)	1509.35 (84.53)	1394.41 (70.88)	1431.24 (93.42)	1513.59 (113.85)	1669.17* (82.93)

Mean (in ms) and standard error (SE) in brackets. Collection latencies were significantly affected by quinpirole [dose: *F*(4,44) = 4.81, *p* = 0.002] with animals being slower at collecting food after administration of the highest dose 1 μg/μl, compared to 0.01 μg/μl (*p* = 0.002) and 0.03 μg/μl (*p* = 0.007). Response latencies were influenced by dose [*F*(4,99) = 7.58, *p* < 0.001] and response type [*F*(1,99) = 241.11, *p* < 0.001]. Rats were faster at making a correct response compared to an incorrect response regardless of dose (*p* < 0.001); however, these responses were significantly slower following administration of the highest dose of quinpirole compared to vehicle and the intermediate doses *p* < 0.05. *p < 0.05 compared to vehicle

## Discussion

The D2/3 receptor agonist, quinpirole, infused into the VTA of rats tested for premature responses on the 5-CSRTT significantly reduced premature responses, though only at the highest dose (1 ug/ul). The rate of reduction of premature responses, across doses of quinpirole, correlated negatively with premature responses in the vehicle condition, suggesting a floor effect of the action of quinpirole in reducing premature responses. These findings are in line with predictions that diminished firing of VTA DA fibres, via activation of D2 somatodendritic autoreceptors, would lead to a decrease in premature responses. As application of quinpirole on midbrain DA cells is known to decrease DA overflow in the NAcb (Anzalone et al. 2012; Schmitz et al. 2002), it is suggested that the decrease in premature responses observed with application of quinpirole 1 ug/ul results from diminished DA efflux onto accumbal neurons, thus confirming a role of mesolimbic DA in anticipatory behaviour. These data are in agreement with the original observations that depletion of mesolimbic DA following intra-accumbens 6-hydroxydopamine reduced premature responding in rats and that the elevation of such responding by intra-accumbens d-amphetamine or by white noise was DA-dependent (Cole and Robbins 1989). The present findings also enrich and refine previous evidence in the laboratory that decreased premature responding observed following systemic administration of quinpirole (0.01, 0.03 and 0.1 mg/kg, Fernando et al. 2012), may result predominantly from changes in midbrain DA-ergic firing. These findings, however, contrast with results of studies investigating effects of quinpirole infused in the NAcb of rats during performance of the 5-CSRTT (Moreno et al. 2013; Pezze et al. 2007). For example, Pezze and colleagues (2007) found that infusions of quinpirole in the NAcb did not significantly affect premature responses (although they did increase perseverative responses), while Moreno and colleagues (2013) found that infusions of quinpirole in the NAcb core (but not the shell) sub-region only increased premature responses in rats with high trait impulsivity but not in low-impulsive rats. The different findings of these studies compared with the present work are likely due to differences in brain region targeted. Thus when infused locally in the NAcb quinpirole will both bind to D2/3 auto-receptors located on VTA terminals projecting to the NAcb as well as to D2/3 receptors located post-synaptically on medium spiny neurons (MSNs) of the NAcb. While the action of quinpirole on the former would presumably cause a reduction in DA release, the effect of the same drug on post-synaptic D2/3 receptors will reduce the firing of D2/3 MSNs, mimicking the effect of DA release onto these cells. The net effect of these two actions clearly results in a behavioural outcome that differs from an action to reduce mesolimbic DA function, as achieved in this present study by local VTA infusion.

In recent years, several lines of evidence have pointed to a role of the NAcb shell sub-region in the production of premature responding in the 5CSRTT. Thus, Diergaarde and colleagues (2008) showed premature responding to be associated with in vitro evidence of increased DA release in the NAcb shell sub-region rather than the core. In addition, high impulsive rats exhibit reduced DA transporters in the shell, but not the core, region (Jupp et al. 2013) and administration of the D3 receptor antagonist nafodotride in the shell enhances premature responding (Besson et al. 2013). Very recently it was shown that increasing the activity of VTA cells, and especially the VTA-shell pathway, in rats, augments the occurrence of premature responses (Flores-Dourojeanni et al. 2021). Thus, altogether, these findings suggest that the core and shell of the NAcb have opponent roles in the regulation of premature responding on the 5CSRTT (see Caprioli et al. 2014; Dalley and Robbins 2017; Diergaarde et al. 2008; Murphy et al. 2008; Sesia et al. 2008). However, this hypothesis cannot be directly tested in the present study due to the difficulty of targeting selectively those VTA DA neurons projecting to the shell region.

It is a limitation of the present investigation that a possible role for dopamine D3 receptors in the VTA cannot be excluded as there is significant though low evidence of D3 receptors in this region. This issue could perhaps be resolved by employing intra-VTA infusions of selective D3 receptor antagonists. Nonetheless, the present investigation is of considerable significance in light of evidence that low D2/3 DA receptor binding in the midbrain of humans, investigated using positron emission tomography, predicted impulsivity scores on the Barratt Impulsiveness Scale and were associated with an increase in amphetamine-induced DA release in the striatum (Buckholtz et al. 2010). This suggests that the present rodent data are relevant to human impulsivity syndromes, potentially including stimulant use disorder (e.g. Dalley et al. 2007).

Finally, in the present study the highest dose, which affected premature responses, did not have any effects on accuracy and reinforcers earned, suggesting that reduction of DA release in the ventral striatum does not affect attentional performance on the 5CSRTT. However, it did significantly lengthen response latency and slightly increased omissions (albeit in the latter case, not significantly), supporting a role of DA in action initiation and speed of responding (Klaus et al. 2019; Mohebi et al. 2019)—and hence in general activation. This is a theoretical construct referring to the vigour of behavioural output, which is also affected by motivational factors consistent with the role of mesolimbic DA in incentive motivation and reward (Wise 2004). Indeed, speed of action is generally considered as an index of motivation (Niv et al. 2007), with more highly motivated animals making faster, more vigorous movements (Mohebi et al. 2019). Premature responses, by definition, are rapidly initiated movements, and so it is possible that the effects of quinpirole to reduce premature responses are due to reduced motivation. Motivational factors have been shown to play a key role in the occurrence of premature responses as shown recently by our group (Toschi et al. 2022) and others (Bizarro and Stolerman 2003; Carli and Samanin 1992; Grottick and Higgins 2000).

In summary, the present data have resolved the likely site of action of systemic quinpirole, in ameliorating premature responding on the 5-CSRTT, to VTA D2/3 autoreceptors on DA neurons projecting to the NAcb. This provides converging evidence linking the mesolimbic dopaminergic system to motor impulsivity. Further investigation should focus on refining, at the cell and circuit-specific level, the mechanisms giving rise to marked individual differences in impulsivity.

## Supplementary Information

Below is the link to the electronic supplementary material.

Supplementary file1 (DOCX 214 KB)
