# Supplementary material for: Effects of quinpirole in the ventral tegmental area on impulsive behaviour during performance on the five-choice serial reaction time task
Source: Exp Brain Res. 2023 Jan 10;241(2):539–46. doi: 10.1007/s00221-022-06502-8 (PMC9895024; doi:10.1007/s00221-022-06502-8)
Supplement: Supplementary file 1 — Supplementary file1 (DOCX 214 KB) [file 221_2022_6502_MOESM1_ESM.docx]

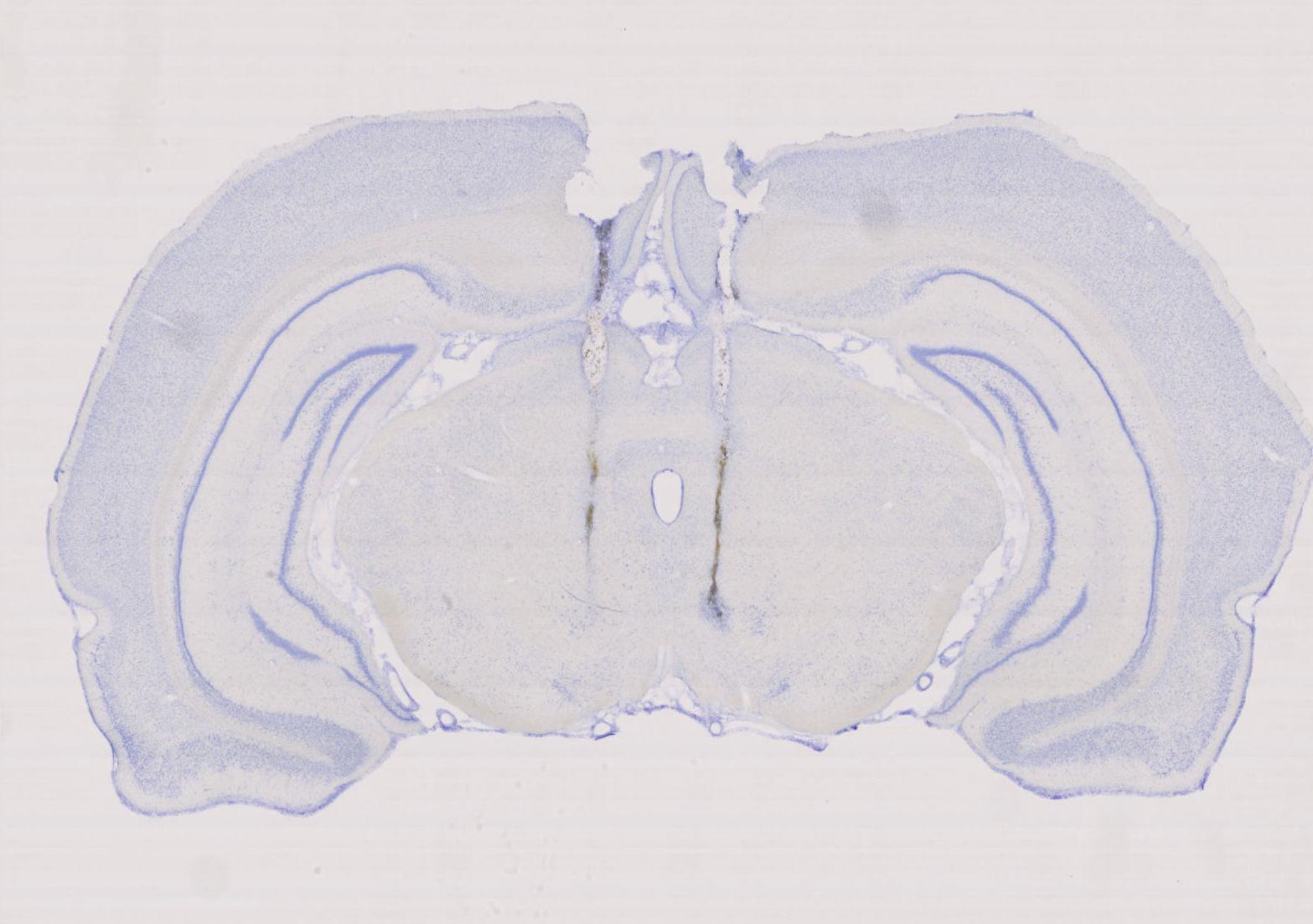


**Figure S1**. Exemplar image of the tract of a cannula implanted in VTA

**Figure S2**. Simple linear regressions (y=mx + c) fitted to each animal showing the change in premature responses as a function of treatment with quinpirole. Veh = vehicle.
